# Comparison of Choledochoduodenostomy and Hepaticogastrostomy for EUS-Guided Biliary Drainage: A Meta-Analysis

**DOI:** 10.3389/fsurg.2022.811005

**Published:** 2022-03-10

**Authors:** Jiasu Li, Jian Tang, Feng Liu, Jun Fang

**Affiliations:** ^1^Department of Gastroenterology, Zhongnan Hospital of Wuhan University, Wuhan, China; ^2^Digestive Endoscopy Center, Shanghai Tenth People's Hospital, Tongji University School of Medicine, Shanghai, China

**Keywords:** biliary drainage, choledochoduodenostomy, hepaticogastrostomy, endoscopic ultrasound, biliary obstruction

## Abstract

**Background:**

Although endoscopic ultrasound-guided choledochoduodenostomy (EUS-CDS) or hepaticogastrostomy (EUS-HGS) has emerged as an option for patients of failed endoscopic retrograde cholangiopancreatography (ERCP), there has no agreement on which approach is preferred. Therefore, a meta-analysis was performed to examine the two methods.

**Methods:**

We performed a comprehensive search in databases of PubMed, Embase, and Cochrane library to find relevant studies reporting the efficacy and safety of the two EUS-guided biliary drainage methods.

**Results:**

In total, 12 studies with 623 patients (EUS-CDS: 303 and EUS-HGS: 320) were included. The cumulative technical success and clinical success for EUS-CDS and EUS-HGS was 95.0% (288/303), 93.1% (268/288), and 96.6% (309/320), 91.3% (282/309), respectively. Compared with EUS-HGS, the pooled odds ratio (OR) was 0.74 (95% CI 0.33–1.65; *p* = 0.46) for EUS-CDS technical success and 0.94 (95% CI 0.56–1.59; *p* = 0.83) for clinical success. The pooled difference in means of procedure time of EUS-CDS and EUS-HGS was −2.68 (95% CI −5.12 to −0.24; *p* = 0.03). The cumulative early adverse events for EUS-CDS and EUS-HGS was 12.2% (37/303) and 17.5% (56/320), respectively. Compared with EUS-HGS, the pooled OR of early adverse events for EUS-CDS was 0.58 (95% CI: 0.36–0.93; *p* = 0.02).

**Conclusion:**

This meta-analysis further suggests EUS-CDS and EUS-HGS have equal high technical and clinical success, but EUS-CDS with a slightly short procedure time and with less early adverse events compared to EUS-HGS.

## Introduction

For obstructive jaundice, Endoscopic retrograde cholangiopancreatography (ERCP)-guided biliary drainage (ERCP-BD), is the preferred choice. For patients with difficult endoscopic biliary drainage, percutaneous transhepatic biliary drainage, or surgery is usually performed. However, these operations are highly invasive and risky of complications. Thus, endoscopic ultrasound-guided biliary drainage (EUS-BD) was developed and showed a promising future. It is reported that EUS-BD could have similar efficacy and safety when compared with ERCP for palliation of distal malignant biliary obstruction and with a lower risk of post-procedure complications (1).

There are two major transgastric/transhepatic or transduodenal routes, namely endoscopic ultrasound-guided choledochoduodenostomy (EUS-CDS) or hepaticogastrostomy (EUS-HGS). Currently, most data show that there is no significant difference in efficacy between EUS-CDS and EUS-HGS or in major complications (2–4). However, some studies have revealed inconsistent conclusions (5, 6), and the optimal method of transluminal biliary drainage has not been established. Therefore, we performed the meta-analysis to examine the two methods.

## Methods

The meta-analysis was conducted under the direction of the Preferred Reporting Items for Systematic Reviews and Meta-Analysis (PRISMA) guidelines and Meta-Analysis of Observational Studies in Epidemiology (MOOSE) guidelines (7, 8).

### Literature Collection

The literature retrieval strategy was based on the PICOS principle (7): P (population): biliary obstruction patients with failed ERCP or were not candidates for ERCP; I (intervention): EUS-CDS and EUS-HGS; C (comparison): efficacy and safety of EUS-CDS and EUS-HGS for biliary drainage; O (outcome): technical and clinical success, operation time, and adverse events; and S (study design): no restriction. PubMed, Embase and Cochrane library were systematically searched from inception through March 2020 to obtain relevant articles. The following keywords were used in various combination: endoscopic ultrasound, biliary drainage, transluminal, choledochoduodenostomy, and hepaticogastrostomy. Additionally, all references in the reviewed articles were manually searched to increase the yield of potentially relevant articles.

### Inclusion and Exclusion Criteria

All the retrieved studies were assessed independently by two researchers. Any disagreement was discussed with a third researcher. Studies that met the following inclusion criteria were considered eligible: (a) study design: randomized clinical trials (RCTs) or observational studies in English language; (b) study population: patients receiving EUS-BD with a sample size >15; and (c) intervention: studies comparing EUS-CDS with EUS-HGS. Exclusion criteria were as follow: (a) republication; (b) animal and review studies; (c) combined with other intervention; and (d) abstracts only and unpublished data.

### Data Collection and Quality Assessment

A standard data collection Excel form was used and performed by two researchers independently. Any disagreement was discussed with a third researcher. The characteristics of the included study were listed as following: first author, year, study design, sample size, adverse events, technical success, clinical success, procedure time, stent patency, and survival days in both groups.

Quality assessment was performed by two authors independently, using the Jadad scale for randomized trials and Newcastle–Ottawa scale (NOS) for observational studies (9, 10). A third author would be consulted and the decision would be reached through discussions when a disagreement was encountered.

### Statistical Analysis

Statistical analysis was conducted using RevMan software (Review Manager Version 5.3; The Nordic Cochrane Centre, Copenhagen, Denmark), with the random effects model for all outcomes. Odds ratio (OR) was calculated for categorical variables (Mantel–Haenszel method) and mean (±SD) or median for continuous variables (Inverse-Variance method) with corresponding 95% CI. Heterogeneity was evaluated by using Cochran's Q-test and *I*^2^ statistics, in which a *p* < 0.10 for Cochran's Q test and the *I*^2^-values > 50% indicated the presence of heterogeneity in this meta-analysis. Funnel plot was generated to test the presence of publication bias and influence of study quality. All *p*-values < 0.05 indicated statistically significance.

## Results

### Study Characteristics

**Figure 1:**
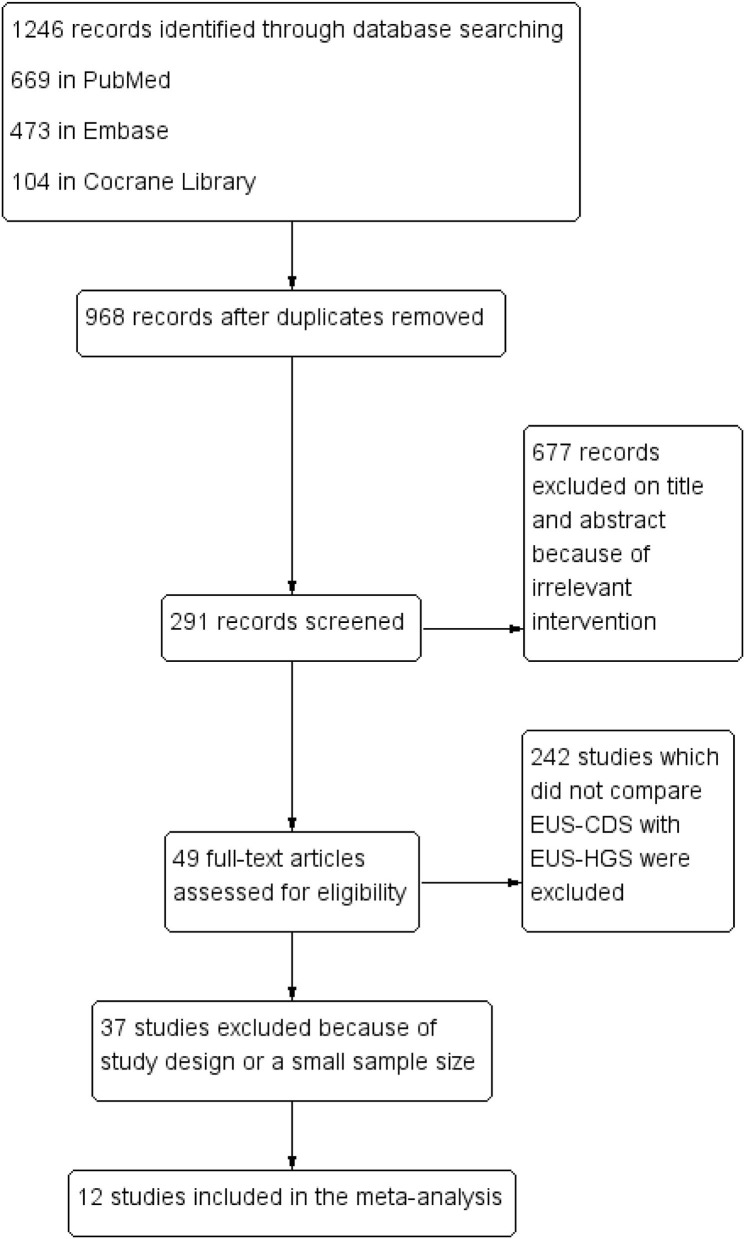
Flow diagram of the study selection process.

According to the pre-defined retrieval strategy, 1,246 abstracts were screened, in which 1,234 were excluded because they did not meet the inclusion criteria. Finally, 12 studies (between 2011 and 2019) involving 623 patients (EUS-CDS: 303 and EUS-HGS: 320) were included in the meta-analysis (6, 11–21). The study screening flow diagram is shown in Figure 1. The primary diagnosis for patients who underwent the procedures were malignant biliary obstructions, and majority of them previously underwent failed ERCP, including due to duodenal obstruction or surgical altered anatomy. Two studies were randomized controlled trials (14, 21), five were prospective uncontrolled studies (11, 13, 15, 19, 20), and five were retrospective studies (6, 12, 16–18). Eight studies were single-center, whereas four studies were multicenter. The characteristics of the eligible studies and the risk of quality assessment are summarized in Table 1.

**Table 1:** The characteristics of included studies.

**Study**	**Type**	**Setting**	**Sample size**		**Technical success**	**Clinical success**	**Mean procedure time, min**	**Mean stent patency, d**	**Early adverse events**	**Late adverse events**	**Median survival time, days**	**Jadad or NOS assessment**
Park et al. (11)	P	S	CDS	26	24	22	16.3 ± 8.8	152	5	4	NR	NOS = 7
			HGS	31	31	27	18.5 ± 9.6	132	6	0	NR	
Kawakubo et al. (12)	R	M	CDS	44	42	41	NR	103	6	9	179 (99–227)	NOS = 6
			HGS	20	19	19	NR	62	6	6	102 (61–262)	
Song et al. (13)	P	S	CDS	17	17	16	22 (14–35)	111 (33–560)	2	1	NR	NOS = 6
			HGS	10	10	10	22.5 (15–35)	181 (36–431)	3	1	NR	
Artifon et al. (14)	RCT	S	CDS	24	22	17	48.8	NR	3	NR	83.59 ± 3.45	Jadad = 5
			HGS	25	24	22	47.8	NR	5	NR	75.08 ± 5.29	
Park et al. (15)	P	M	CDS	12	11	11	10 (9–15)	122 ± 12	1	3	NR	NOS = 6
			HGS	20	20	18	13 (10–21)	121 ± 11.2	5	0	NR	
Poincloux et al. (16)	R	S	CDS	26	26	24	NR	NR	2	3	NR	NOS = 6
			HGS	66	65	61	NR	NR	10	6	NR	
Guo et al. (17)	R	S	CDS	14	14	14	NR	NR	1	NR	NR	NOS = 6
			HGS	7	7	7	NR	NR	2	NR	NR	
Khashab et al. (18)	R	M	CDS	60	56	48	51 ± 34.9	NR	8	8	252 (131–369)	NOS = 7
			HGS	61	56	46	45.3 ± 34.6	NR	12	16	142 (82–256)	
Ogura et al. (6)	R	S	CDS	13	13	13	NR	43	0	6	98	NOS = 6
			HGS	26	26	24	NR	133	0	2	133	
Amano et al. (19)	P	S	CDS	11	11	11	11 (8–16)	NR	2	0	NR	NOS = 7
			HGS	9	9	9	14 (11–18)	NR	1	0	NR	
Cho et al. (20)	P	S	CDS	33	33	33	20 (5–45)	329.1 (231.8–426.4)	5	5	165 (72.2–257.7)	NOS = 7
			HGS	21	21	18	18 (11–45)	166.3 (94.7–237.9)	4	10	173 (76.8–269.1)	
Minaga et al. (21)	RCT	M	CDS	23	19	18	25.2 ± 10.8	NR	2	2	120 (43–408)	Jadad = 6
			HGS	24	21	21	37.7 ± 14.0	306	2	4	146 (21–400)	

*P, prospective; R, retrospective; RCT, randomized controlled trial; S, single-center; M, multicenter; CDS, choledochoduodenostomy; HGS, hepaticogastrostomy; NR, not reported; NOS, Newcastle–Ottawa scale*.

### Study Quality Assessment

The quality of each of the two RCTs was excellent; the two RCTs reported random sequence generation, allocation concealment, and without incomplete outcome data. Although the two RCTs were single-blinded, one RCT reported adequate blinding of outcome assessment (21), so the risk bias of selective reporting of the RCT was considered low risk. In the observational studies, six studies were awarded six stars (6, 12, 13, 15, 16, 18), and the rest were received seven stars (11, 17, 19, 20).

### Meta-Analysis Results

#### Technical and Clinical Success

**Figure 2:**
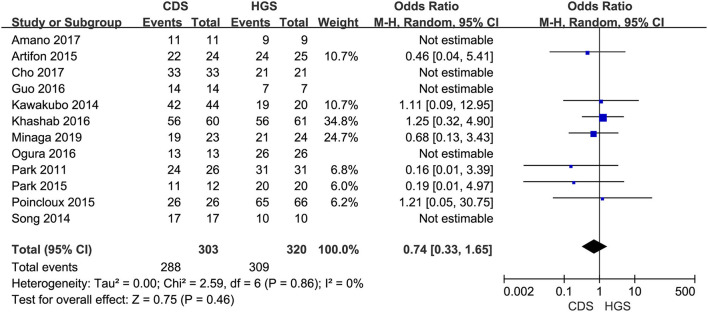
Forest plot of technical success between EUS-CDS and EUS-HGS.

**Figure 3:**
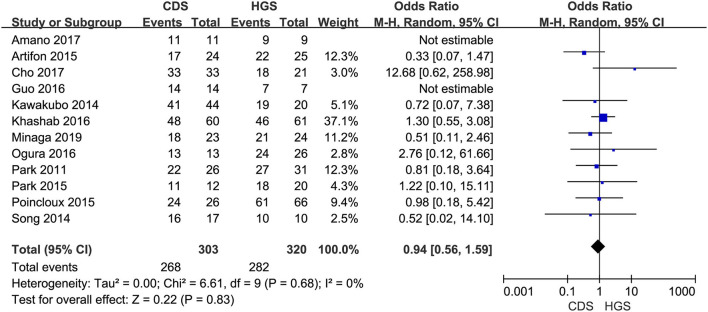
Forest plot of clinical success between EUS-CDS and EUS-HGS.

**Figure 4:**
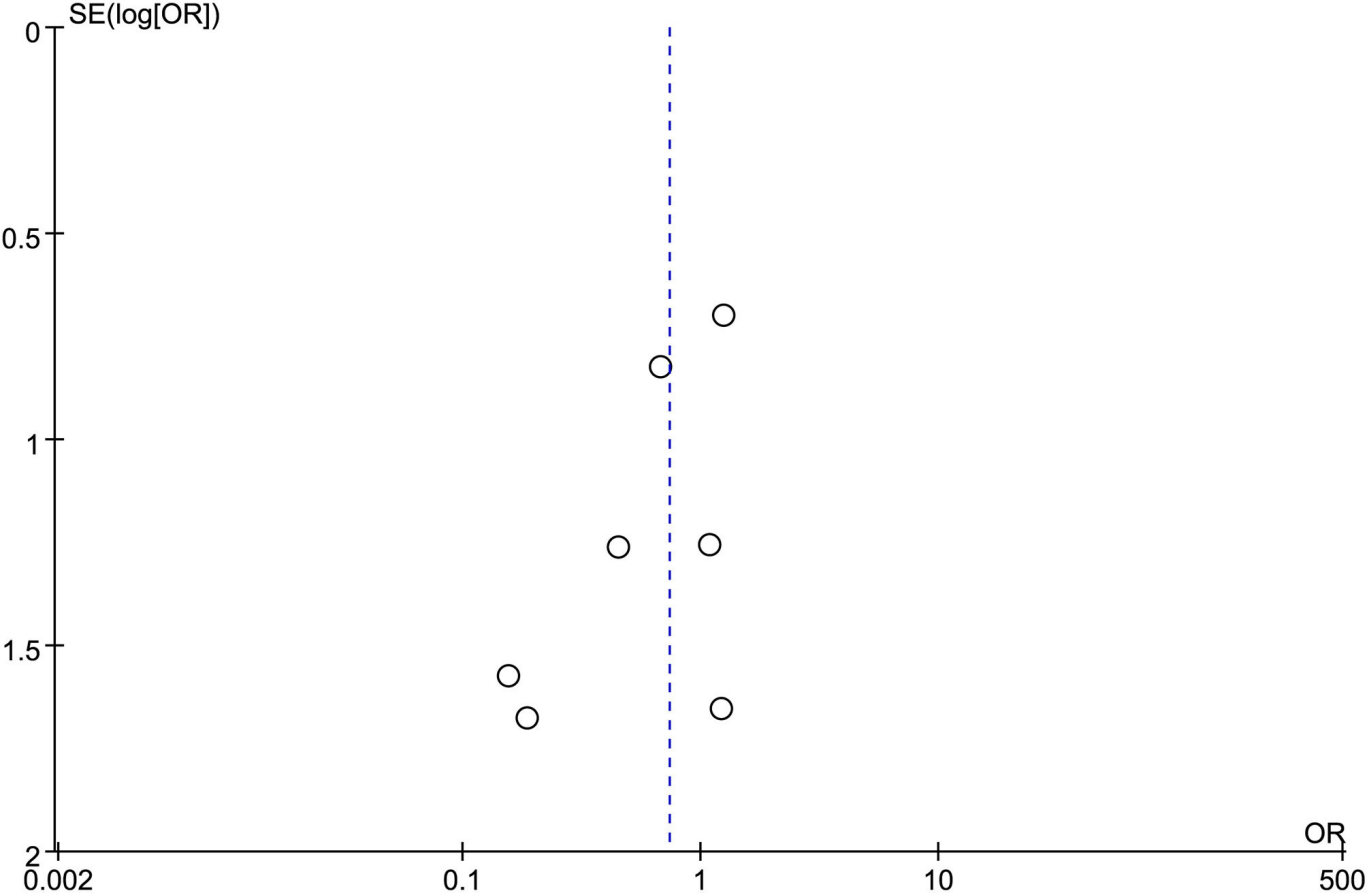
Funnel plot of technical success between EUS-CDS and EUS-HGS.

**Figure 5:**
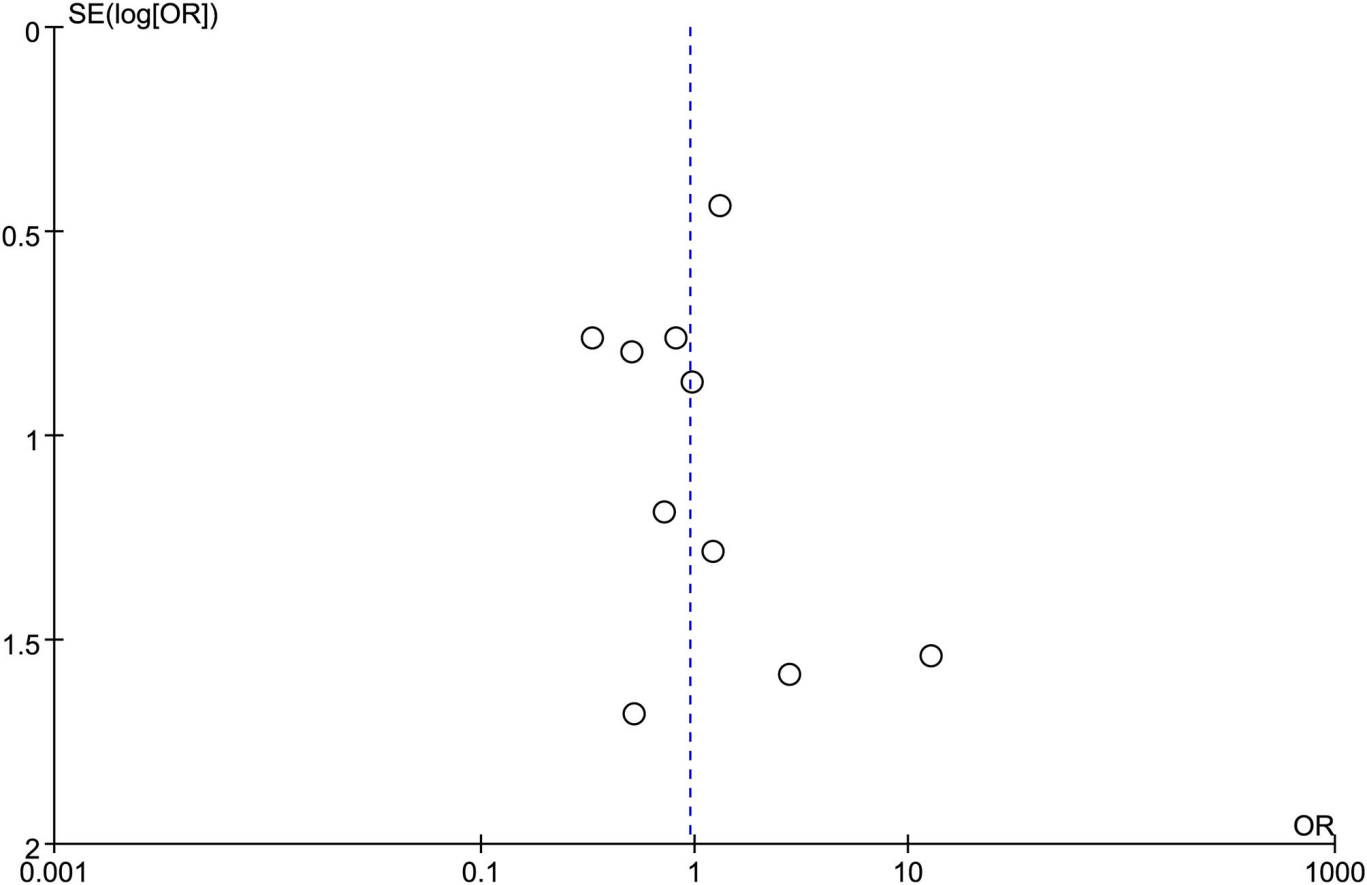
Funnel plot of clinical success between EUS-CDS and EUS-HGS.

All the 12 eligible studies in the analysis reported the technical success and clinical success. The cumulative technical and clinical success rate for EUS-CDS and EUS-HGS was 95.0% (288/303), 93.1% (268/288), and 96.6% (309/320), 91.3% (282/309), respectively. When the first-line EUS-BD procedure failed, another EUS-BD procedure or other interventions, such as EUS-guided gallbladder drainage, were alternatively considered during the same endoscopic session. Compared with EUS-HGS, the pooled OR was 0.74 (95% CI 0.33–1.65; *p* = 0.46) for EUS-CDS technical success and 0.94 (95% CI 0.56–1.59; *p* = 0.83) for clinical success, suggesting no significant difference between CDS and HGS (Figures 2, 3). Heterogeneity was not significant with *I*^2^ = 0% (*p* = 0.86) for technical success and *I*^2^ = 0% (*p* = 0.68) for clinical success. The funnel plot appeared symmetric and without significant publication bias (Figures 4, 5).

#### Procedure Time

**Figure 6:**
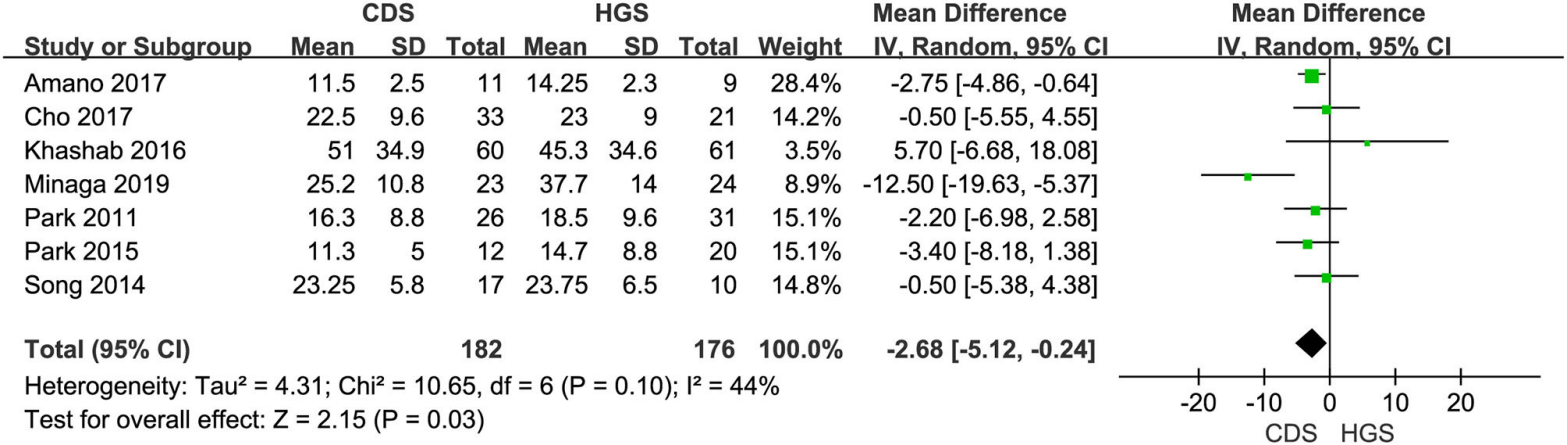
Forest plot of procedure time between EUS-CDS and EUS-HGS.

**Figure 7:**
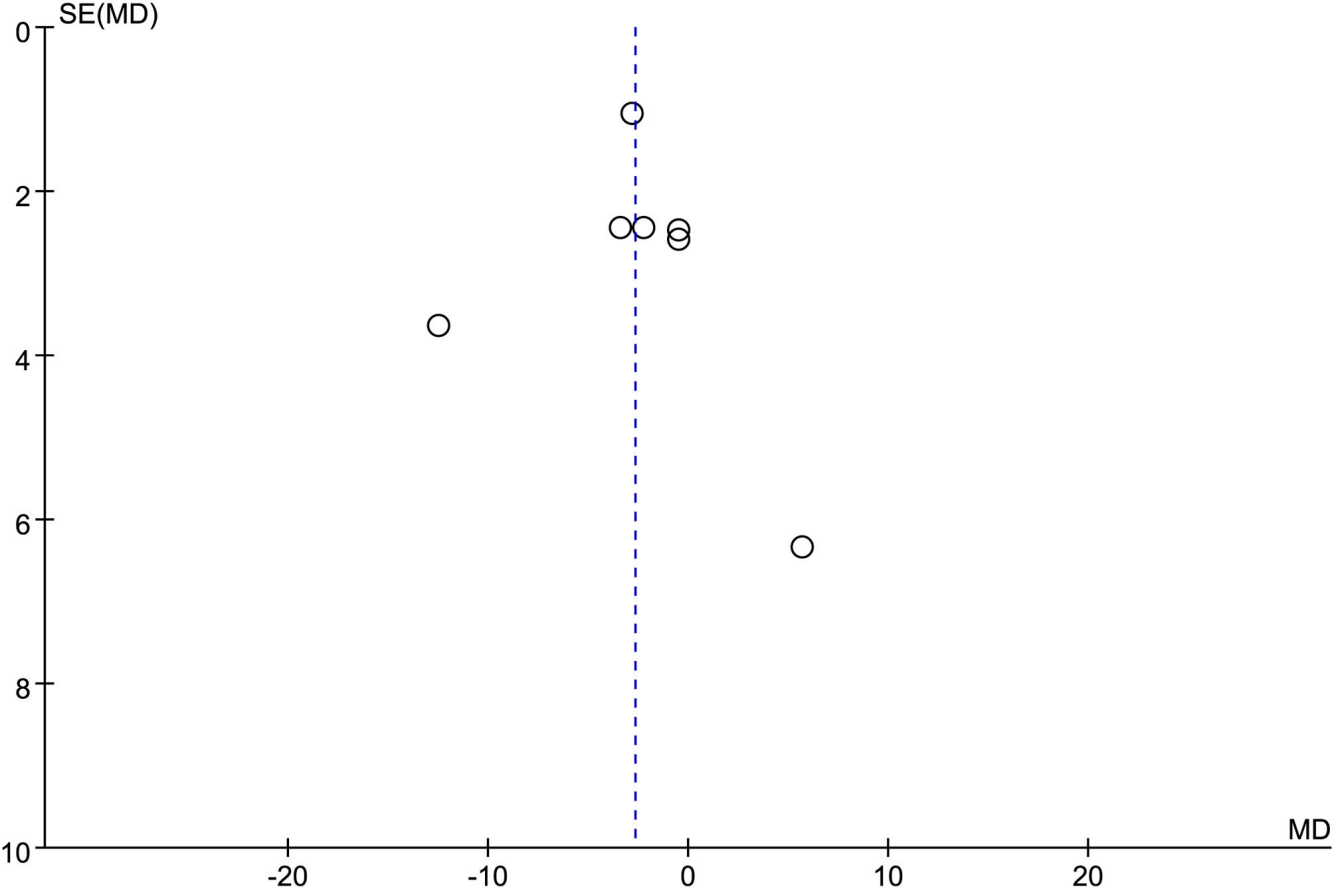
Funnel plot of procedure time between EUS-CDS and EUS-HGS.

**Figure 8:**
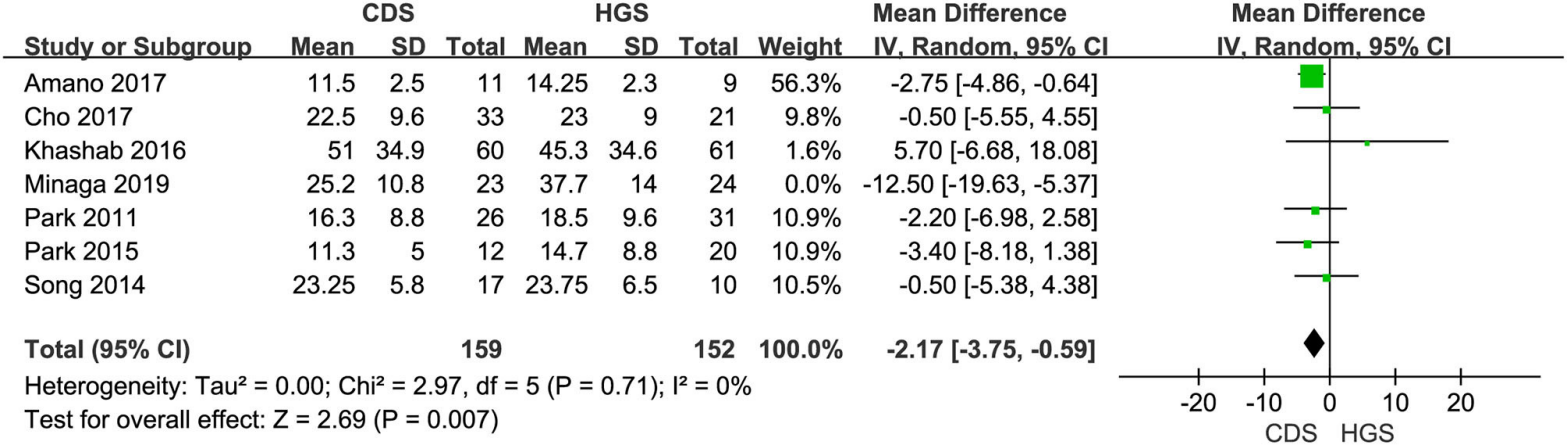
Forest plot of procedure time between EUS-CDS and EUS-HGS (Exclude Minaga's study).

Seven studies were included in this subgroup analysis. Four of them reported data in the mean of median (range/interquartile range) (13, 15, 19, 20), so we estimated the sample mean ± SD from the sample median, range by the statistical methods reported by Luo et al. and Wan et al. (22, 23). The pooled difference in means was −2.68 (95% CI −5.12 to −0.24; *p* = 0.03), and Cochran's Q test (*p* = 0.01) was with moderate heterogeneity (*I*^2^ = 44%), suggesting EUS-CDS was a little faster (Figure 6). The funnel plot of which appeared not symmetric and with a publication bias (Figure 7). The sensitivity analysis by omitting one study at a time showed that, excluded the only RCT study in the subgroup (21), the pooled difference in means of observational studies was −2.17 (95% CI −3.75 to −0.59; *p* = 0.007), Cochran's Q test (*p* = 0.71) without considerable heterogeneity (*I*^2^ = 0%), further indicating EUS-CDS was faster than EUS-HGS (Figure 8). In the sensitivity analysis that used a fixed-effects model, the results were also very similar to those in a random-effects model (data were not shown).

### Adverse Events

**Figure 9:**
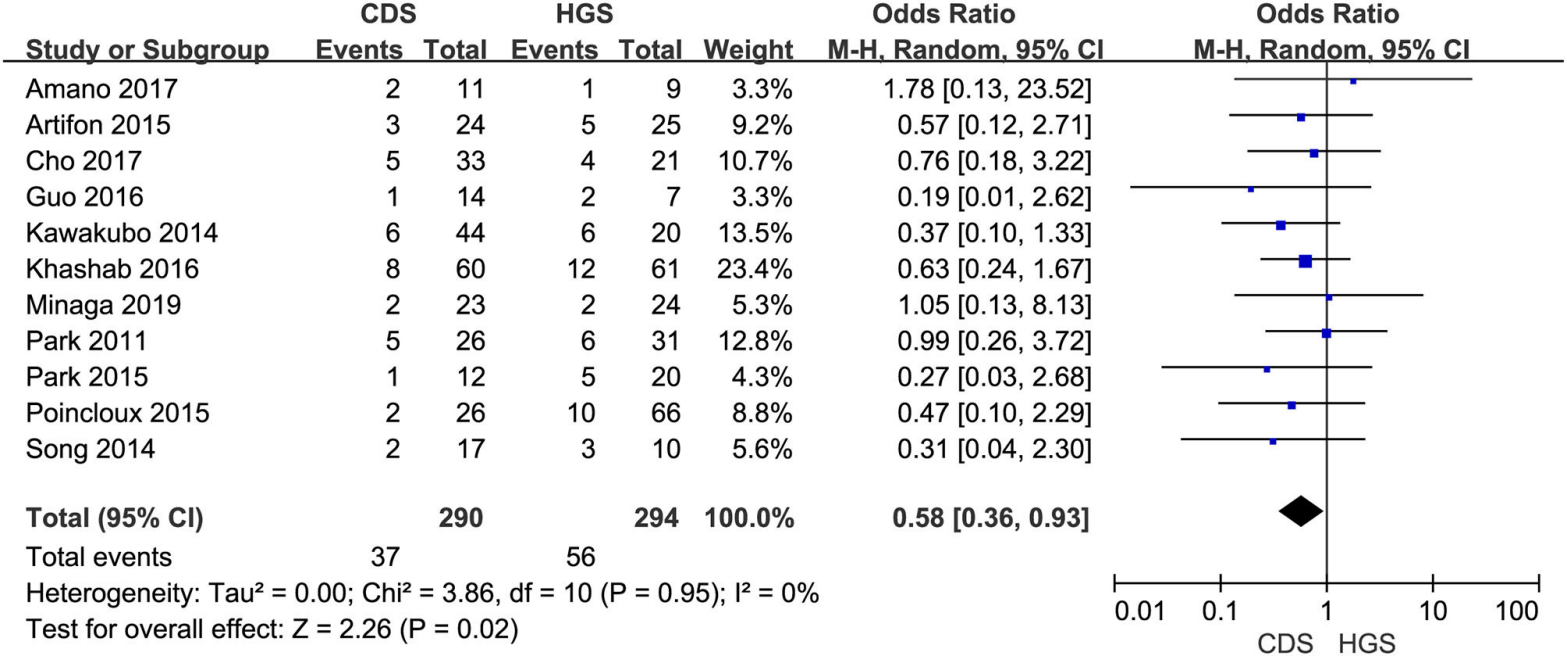
Forest plot of early adverse events between EUS-CDS and EUS-HGS.

**Figure 10:**
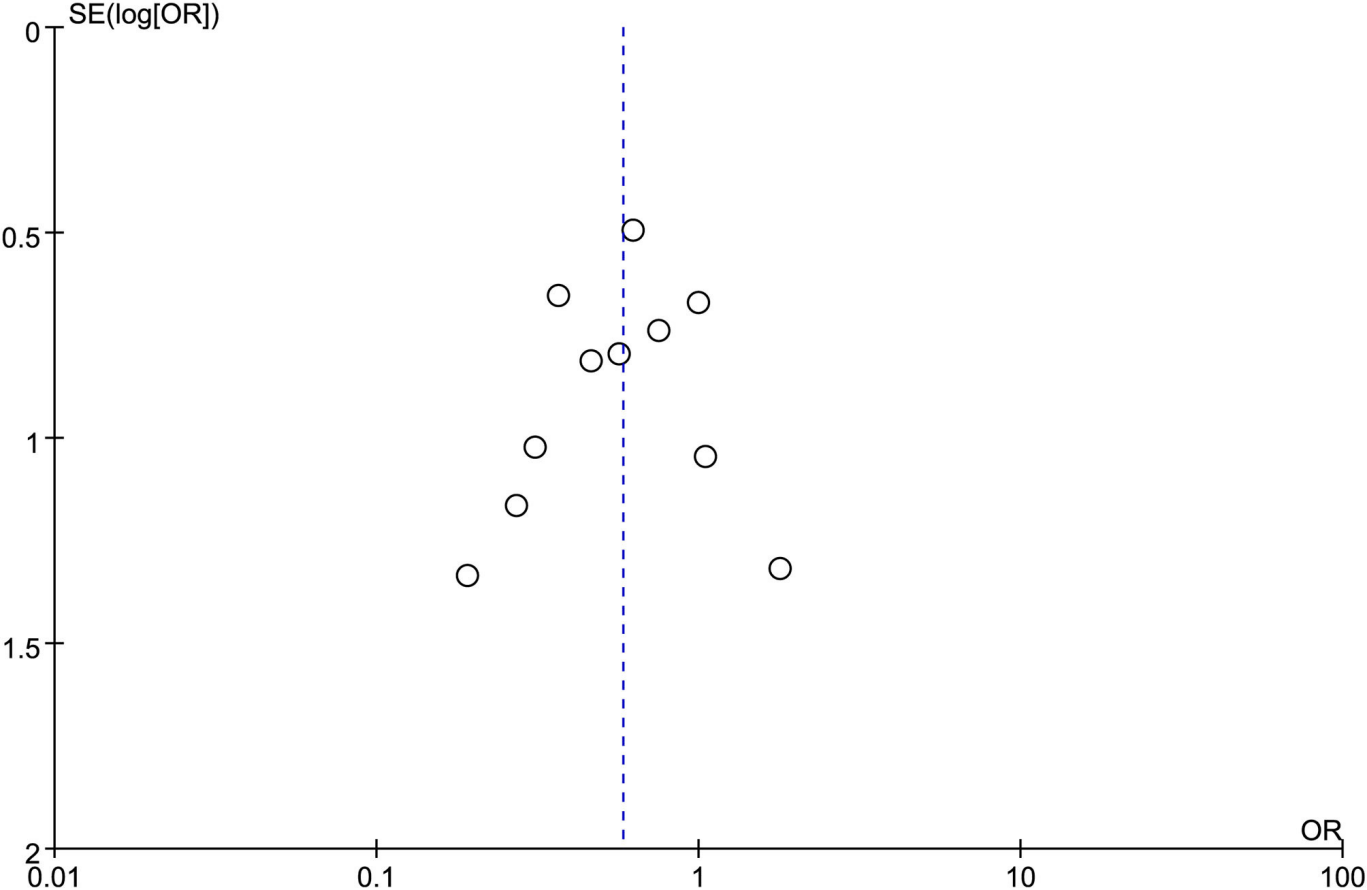
Funnel plot of early adverse events between EUS-CDS and EUS-HGS.

**Figure 11:**
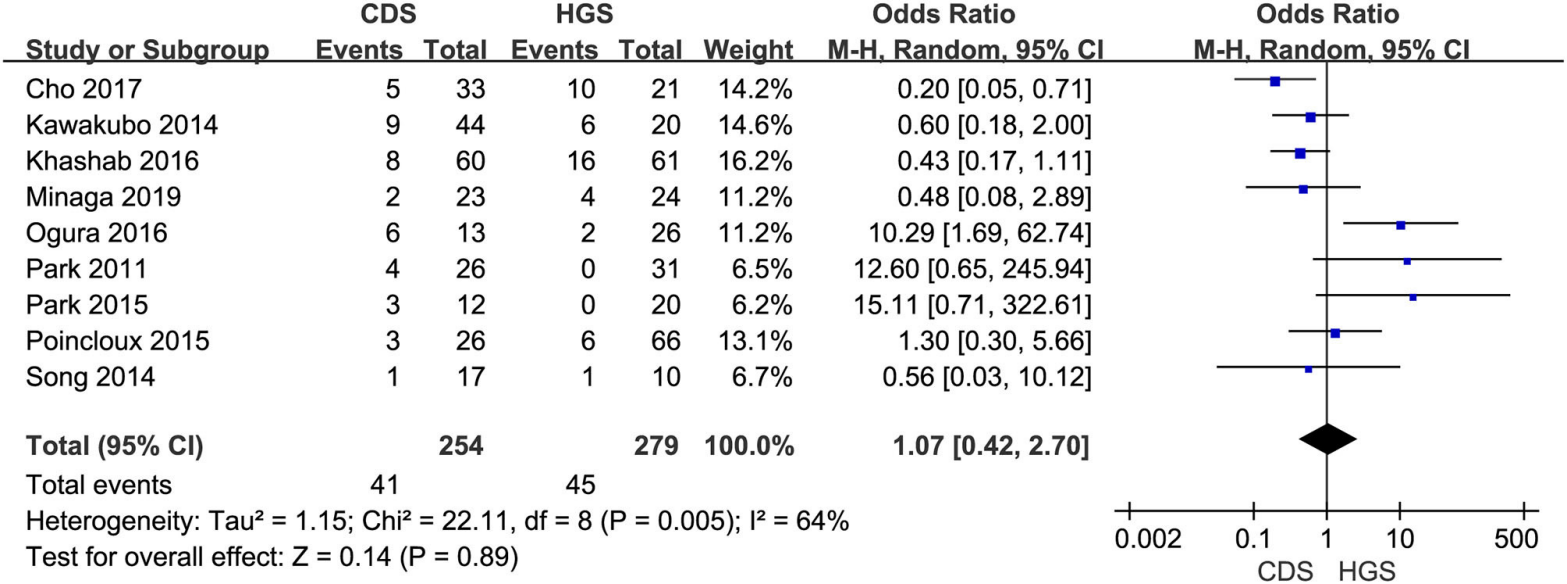
Forest plot of late adverse events between EUS-CDS and EUS-HGS.

**Figure 12:**
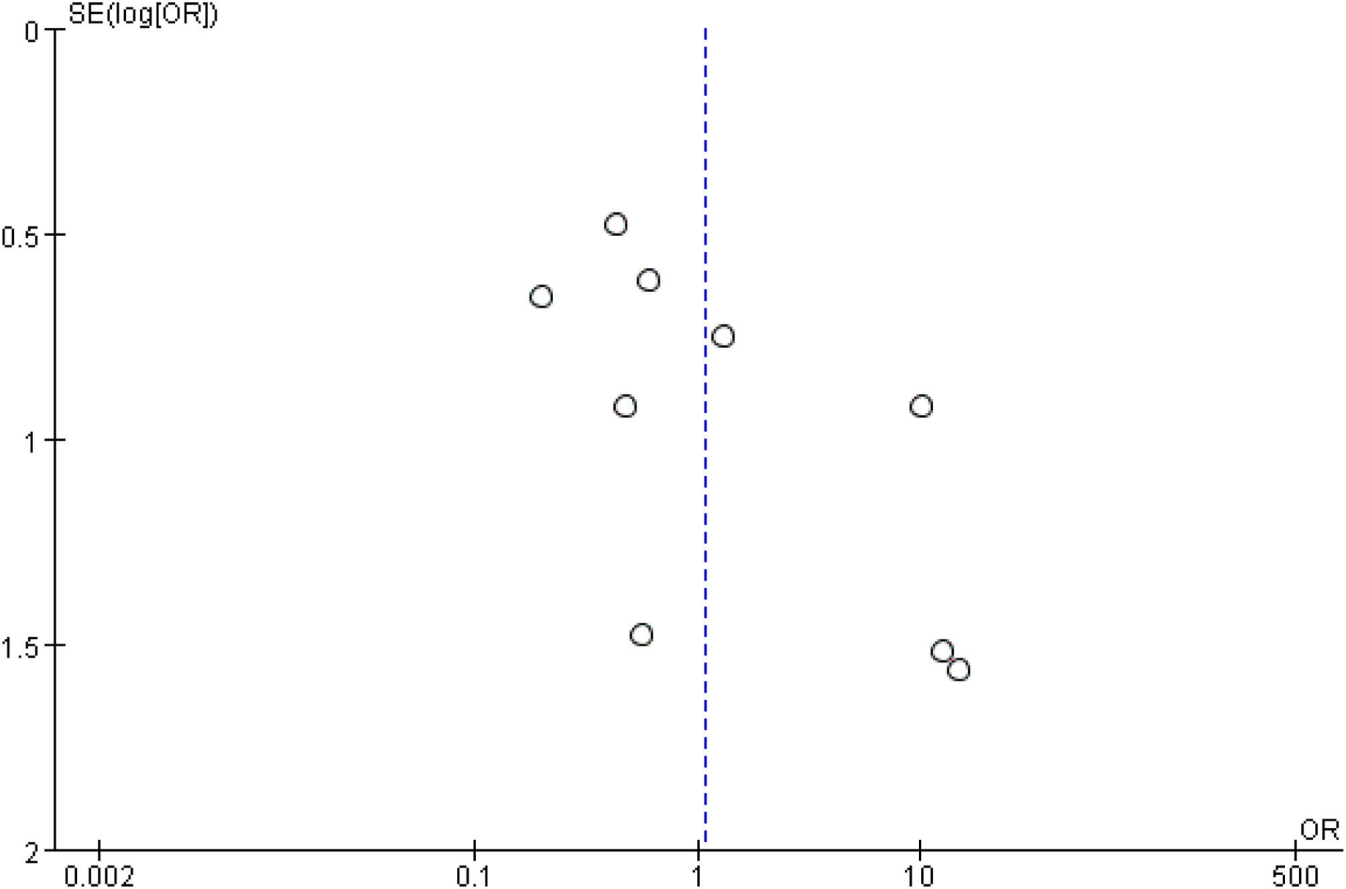
Funnel plot of late adverse events between EUS-CDS and EUS-HGS.

In the eligible studies, the most common adverse events were pneumoperitoneum (*n* = 22), bile leakage/biloma/fistula (*n* = 19), bleeding/hematoma (*n* = 14), cholangitis/sepsis (*n* = 14), and biliary peritonitis (*n* = 8). Eleven studies reported early adverse events. The cumulative early adverse events of EUS-CDS and EUS-HGS were 12.2% (37/303) and 17.5% (56/320), respectively. Pooled OR for early adverse events was 0.58 (95% CI: 0.36–0.93; *p* = 0.02), Cochran's Q test (*p* = 0.95) with no heterogeneity (*I*^2^ = 0%), indicating that EUS-CDS was safer than that of EUS-HGS (Figure 9). The funnel plot of which appeared symmetric and without significant publication bias (Figure 10). Nine studies reported late adverse events, mainly stent occlusion/migration (11–13, 15–18, 20, 21). Pooled OR for late adverse events was 1.07 (95% CI: 0.42–2.70; *p* = 0.89), but the result was limited by considerable heterogeneity (*I*^2^ = 64%, *p* = 0.005; Figure 11). The funnel plot of which appeared not symmetric and with a publication bias (Figure 12). We analyzed some early adverse events separately, but there was no difference in risk of pneumoperitoneum (*p* = 0.28), bile leakage/biloma/fistula (*p* = 0.09), bleeding/hematoma (*p* = 0.11), cholangitis/sepsis (*p* = 0.55), and biliary peritonitis (*p* = 0.45).

## Discussion

This meta-analysis further suggested EUS-CDS and EUS-HGS have equal high technical and clinical success rate, whereas EUS-CDS with a slightly short procedure time, which was in line with a previous meta-analysis (3). Moreover, this meta-analysis found EUS-CDS with a less frequent early adverse events and might be a safer approach compared to EUS-HGS.

The EUS-BD can be performed by direct transluminal stenting using either CDS or HGS, EUS-assisted rendezvous (EUS-RV), and antegrade transpapillary (or transanastomotic) stent placement. Available literatures have reported promising results (24). EUS-BD has primarily been used as rescue therapy after failed ERCP. EUS-BD enables direct access to the bile ducts from either the stomach or the duodenum route without the need to reach the papilla. The optimal drainage strategy depends on the patient's anatomy, the underlying disease (benign/malignant), the location of the obstruction (distal/hilar), and operator expertise (24).

The previous first meta-analysis included 10 studies with 434 patients and did not demonstrate any superiority in terms of efficacy and safety (pooled OR = 0.97, *p* = 0.90) of EUS-CDS and EUS-HGS (3). The above meta-analysis was limited by including small sample size studies. The other meta-analysis was a proportion meta-analysis, including only 5 studies described cohorts of both CDS and HGS, showed more complications with EUS-HGS than EUS-CDS (pooled OR = 2.01, *p* = 0.0042), but most of complications were cases of stent dysfunction (25). A fewer number of included studies, a smaller sample size, and different eligibility criteria might explain the different result. A multicenter retrospective analysis reported that the transhepatic access was the only independent risk factor for adverse events (*p* = 0.031) (5), but the study was excluded from the meta-analysis because of not a dedicated comparison of EUS-CDS with EUS-HGS.

In this meta-analysis, 12 studies dedicated comparison of EUS-CDS with EUS-HGS for biliary drainage, and the total sample size was greater than before, including a recent RCT study (21). Most studies defined adverse events as early and late groups. Early adverse events included pneumoperitoneum, biliary peritonitis, bleeding, bile leakage, abdominal pain, perforation, and cholangitis, whereas the stent dysfunction (stent occlusion/migration) was defined as the late adverse event. Because of the different eligibility criteria and the heterogeneity in the definition of late adverse events of the included studies, we found more early but not late (namely stent dysfunction) complications with EUS-HGS than EUS-CDS, suggesting EUS-CDS might be safer than EUS-HGS. Of course, the late adverse events were more related with the type of stent used in the included studies, which we could not discussed in detail due to the natural limitations of the including studies.

Available literatures have showed that the overall adverse events rate of CDS was 13.6–20%, including duodenal bleeding, bile leak, stent dysfunction, peritonitis, pneumoperitoneum, and cholangitis (26) and was 18% for HGS, including abdominal pain, self-limiting pneumoperitoneum, bile leak, cholangitis, and bleeding (27). Most of them can be managed endoscopically or through interventional radiology. For beginners, EUS-HGS has more types of adverse events than EUS-CDS. Furthermore, EUS-CDS has been more widely used because the site of puncture for EUS is relatively closer and technically easier (27). Therefore, EUS-HGS should be tried after sufficient experience of EUS-guided tissue acquisition, pseudocyst drainage, and EUS-CDS, and it is important to reduce the number of accessory changes and to shorten the procedure time.

It was reported that EUS-HGS had longer stent patency than EUS-CDS in duodenal obstruction patients (median 133 vs. 37 days, *p* = 0.045) (6), which was contrary to a recent multicenter trial that showed there was no significant difference in stent patency between HGS and CDS (28). In this meta-analysis, we could not compare the stent patency between CDS and HGS because of the heterogeneity of the type of stent and the data provided by the included studies. But on the other hand, it was reported that the type of stent used in the procedure might be more significant for stent patency. For example, to weaken the risk of stent dysfunction or migration, a lumen-apposing metal stent (LAMS) is being increasingly used for transmural biliary drainage. A recent meta-analysis concluded EUS-CDS using LAMS was effective and safe for distal biliary obstruction patients (29). The long-term results of EUS-HGS with a long, partially covered metal stent for unresectable malignant biliary obstruction were also safe and effective (30). The type and length of stent are important considerations, as it is stated by a multi-institution consensus, during the transgastric–transhepatic approach, a longer self-expandable metal stent (SEMS, 8 or 10 cm) is recommended, whereas during the transduodenal–transcholedochal approach, a 6-cm SEMS is recommended (4). Further high-quality studies are required to assess CDS and HGS using stent in the same setting for biliary drainage.

There are several limitations in the current meta-analysis. Only two of the eligible studies were randomized studies. The definitions of early and late adverse events were not uniform, leading to a moderate heterogeneity in the analyses, but the sensitivity analysis showed a stable result. Due to the limitations of the retrospective study design, some data were missing in the studies used for meta-analysis. So, we could not get enough data to include stent type and stent patency rate as parameters for meta-analysis. Prospective multicenter cohorts are needed to clarify these issues.

In conclusion, this meta-analysis further suggests EUS-CDS and EUS-HGS have equal high technical and clinical success rate, whereas EUS-CDS with a slightly short procedure time. Furthermore, EUS-CDS has less frequent early adverse events and might be a safer approach compared to EUS-HGS. Due to the limitations of included studies, further high-quality studies are required to confirm these findings and further compare the 2 routes.

## Data Availability Statement

The original contributions presented in the study are included in the article/supplementary material, further inquiries can be directed to the corresponding authors.

## Author Contributions

JF and FL conceived and designed the study. JL and JT selected the studies, collected the data, and analyzed the data. JL drafted the manuscript. All authors interpreted the results, revised the draft manuscript, read, and approved the final version of the manuscript.

## Funding

This study was supported by grants from the Health Commission of Hubei Province (Nos. WJ2019M213 and ZLYNXM202017).

## Conflict of Interest

The authors declare that the research was conducted in the absence of any commercial or financial relationships that could be construed as a potential conflict of interest.

## Publisher's Note

All claims expressed in this article are solely those of the authors and do not necessarily represent those of their affiliated organizations, or those of the publisher, the editors and the reviewers. Any product that may be evaluated in this article, or claim that may be made by its manufacturer, is not guaranteed or endorsed by the publisher.

## References

[1] Jin Z Wei Y Lin H Huang H Lv W Zhang X. Endoscopic ultrasound versus endoscopic retrograde cholangiopancreatography-guided biliary drainage for primary decompression of malignant biliary obstruction: protocol for a systematic review and meta-analysis of randomised controlled trials. BMJ Open. (2019) 9:e028156. 10.1136/bmjopen-2018-028156 31203246 PMC6588990

[2] Wang K Zhu J Xing L Wang Y Jin Z Li Z. Assessment of efficacy and safety of EUS-guided biliary drainage: a systematic review. Gastrointest Endosc. (2016) 83:1218–27. 10.1016/j.gie.2015.10.033 26542374

[3] Uemura RS Khan MA Otoch JP Kahaleh M Montero EF Artifon ELA. EUS-guided choledochoduodenostomy versus hepaticogastrostomy: a systematic review and meta-analysis. J Clin Gastroenterol. (2018) 52:123–30. 10.1097/MCG.0000000000000948 29095426

[4] Guo J Giovannini M Sahai AV Saftoiu A Dietrich CF Santo E . A multi-institution consensus on how to perform EUS-guided biliary drainage for malignant biliary obstruction. Endosc Ultrasound. (2018) 7:356–65. 10.4103/eus.eus_53_18 30531022 PMC6289007

[5] Dhir V Artifon EL Gupta K Vila JJ Maselli R Frazao M . Multicenter study on endoscopic ultrasound-guided expandable biliary metal stent placement: choice of access route, direction of stent insertion, and drainage route. Dig Endosc. (2014) 26:430–5. 10.1111/den.12153 23941261

[6] Ogura T Chiba Y Masuda D Kitano M Sano T Saori O . Comparison of the clinical impact of endoscopic ultrasound-guided choledochoduodenostomy and hepaticogastrostomy for bile duct obstruction with duodenal obstruction. Endoscopy. (2016) 48:156–63. 10.1055/s-0034-1392859 26382307

[7] Liberati A Altman DG Tetzlaff J Mulrow C Gøtzsche PC Ioannidis JP . The PRISMA statement for reporting systematic reviews and meta-analyses of studies that evaluate healthcare interventions: explanation and elaboration. BMJ. (2009) 339:b2700. 10.1136/bmj.b2700 19622552 PMC2714672

[8] Stroup DF Berlin JA Morton SC Olkin I Williamson GD Rennie D . Meta-analysis of observational studies in epidemiology: a proposal for reporting. meta-analysis of observational studies in epidemiology (MOOSE) group. JAMA. (2000) 283:2008–12. 10.1001/jama.283.15.2008 10789670

[9] Jadad AR Moore RA Carroll D Jenkinson C Reynolds DJ Gavaghan DJ . Assessing the quality of reports of randomized clinical trials: is blinding necessary? Control Clin Trials. (1996) 17:1–12. 10.1016/0197-2456(95)00134-4 8721797

[10] Stang A. Critical evaluation of the Newcastle-Ottawa scale for the assessment of the quality of nonrandomized studies in meta-analyses. Eur J Epidemiol. (2010) 25:603–5. 10.1007/s10654-010-9491-z 20652370

[11] Park DH Jang JW Lee SS Seo DW Lee SK Kim MH. EUS-guided biliary drainage with transluminal stenting after failed ERCP: predictors of adverse events and long-term results. Gastrointest Endosc. (2011) 74:1276–84. 10.1016/j.gie.2011.07.054 21963067

[12] Kawakubo K Isayama H Kato H Itoi T Kawakami H Hanada K . Multicenter retrospective study of endoscopic ultrasound-guided biliary drainage for malignant biliary obstruction in Japan. J Hepatobiliary Pancreat Sci. (2014) 21:328–34. 10.1002/jhbp.27 24026963

[13] Song TJ Lee SS Park DH Seo DW Lee SK Kim MH. Preliminary report on a new hybrid metal stent for EUS-guided biliary drainage (with videos). Gastrointest Endosc. (2014) 80:707–11. 10.1016/j.gie.2014.05.327 25053527

[14] Artifon EL Marson FP Gaidhane M Kahaleh M Otoch JP. Hepaticogastrostomy or choledochoduodenostomy for distal malignant biliary obstruction after failed ERCP: is there any difference? Gastrointest Endosc. (2015) 81:950–9. 10.1016/j.gie.2014.09.047 25500330

[15] Park DH Lee TH Paik WH Choi JH Song TJ Lee SS . Feasibility and safety of a novel dedicated device for one-step EUS-guided biliary drainage: a randomized trial. J Gastroenterol Hepatol. (2015) 30:1461–6. 10.1111/jgh.13027 26146796

[16] Poincloux L Rouquette O Buc E Privat J Pezet D Dapoigny M . Endoscopic ultrasound-guided biliary drainage after failed ERCP: cumulative experience of 101 procedures at a single center. Endoscopy. (2015) 47:794–801. 10.1055/s-0034-1391988 25961443

[17] Guo J Sun S Liu X Wang S Ge N Wang G. Endoscopic ultrasound-guided biliary drainage using a fully covered metallic stent after failed endoscopic retrograde cholangiopancreatography. Gastroenterol Res Pract. (2016) 2016:9469472. 10.1155/2016/9469472 27594881 PMC4983388

[18] Khashab MA Messallam AA Penas I Nakai Y Modayil RJ De la Serna C . International multicenter comparative trial of transluminal EUS-guided biliary drainage via hepatogastrostomy vs choledochoduodenostomy approaches. Endosc Int Open. (2016) 4:E175–81. 10.1055/s-0041-109083 26878045 PMC4751013

[19] Amano M Ogura T Onda S Takagi W Sano T Okuda A . Prospective clinical study of endoscopic ultrasound-guided biliary drainage using novel balloon catheter (with video). J Gastroenterol Hepatol. (2017) 32:716–20. 10.1111/jgh.13489 27420770

[20] Cho DH Lee SS Oh D Song TJ Park DH Seo DW . Long-term outcomes of a newly developed hybrid metal stent for EUS-guided biliary drainage (with videos). Gastrointest Endosc. (2017) 85:1067–75. 10.1016/j.gie.2016.09.010 27650270

[21] Minaga K Ogura T Shiomi H Imai H Hoki N Takenaka M . Comparison of the efficacy and safety of endoscopic ultrasound-guided choledochoduodenostomy and hepaticogastrostomy for malignant distal biliary obstruction: Multicenter, randomized, clinical trial. Dig Endosc. (2019) 31:575–82. 10.1111/den.13406 30908711

[22] Luo D Wan X Liu J Tong T. Optimally estimating the sample mean from the sample size, median, mid-range, and/or mid-quartile range. Stat Methods Med Res. (2018) 27:1785–805. 10.1177/0962280216669183 27683581

[23] Wan X Wang W Liu J Tong T. Estimating the sample mean and standard deviation from the sample size, median, range and/or interquartile range. BMC Med Res Methodol. (2014) 14:135. 10.1186/1471-2288-14-135 25524443 PMC4383202

[24] van der Merwe SW van Wanrooij RLJ Bronswijk M Everett S Lakhtakia S Rimbas M . Therapeutic endoscopic ultrasound: European society of gastrointestinal endoscopy (ESGE) guideline. Endoscopy. (2021) 54:185–205. 10.1055/a-1717-1391 34937098

[25] Hedjoudje A Sportes A Grabar S Zhang A Koch S Vuitton L . Outcomes of endoscopic ultrasound-guided biliary drainage: a systematic review and meta-analysis. United European Gastroenterol J. (2019) 7:60–8. 10.1177/2050640618808147 30788117 PMC6374841

[26] Artifon ELA Visconti TAC Brunaldi VO. Choledochoduodenostomy: outcomes and limitations. Endosc Ultrasound. (2019) 8:S72–8. 10.4103/eus.eus_62_19 31897383 PMC6896435

[27] Paik WH Park DH. Outcomes and limitations: EUS-guided hepaticogastrostomy. Endosc Ultrasound. (2019) 8:S44–9. 10.4103/eus.eus_51_19 31897379 PMC6896431

[28] Paik WH Lee TH Park DH Choi JH Kim SO Jang S . EUS-guided biliary drainage versus ERCP for the primary palliation of malignant biliary obstruction: a multicenter randomized clinical trial. Am J Gastroenterol. (2018) 113:987–97. 10.1038/s41395-018-0122-8 29961772

[29] Krishnamoorthi R Dasari CS Thoguluva Chandrasekar V Priyan H Jayaraj M Law J . Effectiveness and safety of EUS-guided choledochoduodenostomy using lumen-apposing metal stents (LAMS): a systematic review and meta-analysis. Surg Endosc. (2020) 34:2866–77. 10.1007/s00464-020-07484-w 32140862

[30] Nakai Y Sato T Hakuta R Ishigaki K Saito K Saito T . Long-term outcomes of a long, partially covered metal stent for EUS-guided hepaticogastrostomy in patients with malignant biliary obstruction (with video). Gastrointest Endosc. (2020) 92:623–31. 10.1016/j.gie.2020.03.3856 32278705

